# Efficient bioconversion of raspberry ketone in *Escherichia coli* using fatty acids feedstocks

**DOI:** 10.1186/s12934-021-01551-0

**Published:** 2021-03-12

**Authors:** Chen Chang, Bo Liu, Yihong Bao, Yong Tao, Weifeng Liu

**Affiliations:** 1 College of Forestry, Northeast Forestry University, No. 26 Hexing Road, Harbin, Heilongjiang Province 150040 PR China; 2 CAS Key Laboratory of Microbial Physiological and Metabolic Engineering, State Key Laboratory of Microbial Resources, Institute of Microbiology, Chinese Academy of Sciences, NO. 1 Beichen West Road, Chaoyang District, Beijing, 100101 PR China; 3 University of Chinese Academy of Sciences, Shijingshan District, NO. 19A Yuquan Road, Beijing, 100049 PR China; 4 Heilongjiang Key Laboratory of Forest Food Resources Utilization, No. 26 Hexing Road, Harbin, Heilongjiang Province 150040 PR China

**Keywords:** Raspberry ketone, Fatty acids feedstock, Bioconversion, Phenylpropanoids, *Escherichia coli*

## Abstract

**Background:**

Phenylpropanoid including raspberry ketone, is a kind of important natural plant product and widely used in pharmaceuticals, chemicals, cosmetics, and healthcare products. Bioproduction of phenylpropanoid in *Escherichia coli* and other microbial cell factories is an attractive approach considering the low phenylpropanoid contents in plants. However, it is usually difficult to produce high titer phenylpropanoid production when fermentation using glucose as carbon source. Developing novel bioprocess using alternative sources might provide a solution to this problem. In this study, typical phenylpropanoid raspberry ketone was used as the target product to develop a biosynthesis pathway for phenylpropanoid production from fatty acids, a promising alternative low-cost feedstock.

**Results:**

A raspberry ketone biosynthesis module was developed and optimized by introducing 4-coumarate-CoA ligase (4CL), benzalacetone synthase (BAS), and raspberry ketone reductase (RZS) in *Escherichia coli* strains CR1–CR4. Then strain CR5 was developed by introducing raspberry ketone biosynthesis module into a fatty acids-utilization chassis FA09 to achieve production of raspberry ketone from fatty acids feedstock. However, the production of raspberry ketone was still limited by the low biomass and unable to substantiate whole-cell bioconversion process. Thus, a process by coordinately using fatty-acids and glycerol was developed. In addition, we systematically screened and optimized fatty acids-response promoters. The optimized promoter Pfrd3 was then successfully used for the efficient expression of key enzymes of raspberry ketone biosynthesis module during bioconversion from fatty acids. The final engineered strain CR8 could efficiently produce raspberry ketone repeatedly using bioconversion from fatty acids feedstock strategy, and was able to produce raspberry ketone to a concentration of 180.94 mg/L from soybean oil in a 1-L fermentation process.

**Conclusion:**

Metabolically engineered *Escherichia coli* strains were successfully developed for raspberry ketone production from fatty acids using several strategies, including optimization of bioconversion process and fine-tuning key enzyme expression. This study provides an essential reference to establish the low-cost biological manufacture of phenylpropanoids compounds.

**Supplementary Information:**

The online version contains supplementary material available at 10.1186/s12934-021-01551-0.

## Background

Major industrial biotechnological effort is focused on developing efficient bioproduction routes to high value-added chemicals that are more cost-effective than conventional petrochemical routes [1, 2]. Therefore, increasing important bioprocess metrics, such as titer, yield of target products, and using cheaper raw materials, have become central to metabolic engineering [3, 4]. Phenylpropanoids, including flavonoids, are a diverse family of compounds mainly synthesized by plants from aromatic amino acids phenylalanine and tyrosine [5]. Phenylpropanoids are among the most important natural products and possess diverse important functions with applications in various fields, such as pharmaceuticals, food, cosmetics, and healthcare products [6–8]. However, the low phenylpropanoid contents in plants limit the use of plant extraction routes for production [9, 10]. Recently, phenylpropanoid production by microbial cell factories has attracted significant attention [11, 12].

Typical phenylpropanoid molecules contain an aromatic phenyl group, and their biosynthesis usually requires coumaroyl-coenzyme A (CoA) and several molecules of malonyl-CoA as precursors. Raspberry ketone (RK; 4-(4-hydroxyphenyl)-2-butanone) is the simplest phenylpropanoid and has been wildly used in the fields of cosmetics, food additive. Biosynthesis of raspberry ketone requires one coumaroyl-coenzyme A (CoA) and one malonyl-CoA as precursors. In previous studies, metabolic engineering for raspberry ketone and other phenylpropanoids productions, such as naringenin and curcumin, has been conducted in *E. coli*, yeast, and other microbial factories [13–15]. Major approach to metabolic engineering for raspberry ketone and other phenylpropanoids production involves introducing key enzyme responses to target metabolic pathways. 4-Coumaroyl-CoA is generated from 4-coumaric acid under catalysis by 4-coumaroyl CoA ligase (4CL) [16]. 4-Coumaroyl-CoA is then condensed with several malonyl-CoA extender units under catalysis by chalcone synthase (CHS) to yield chalcone. Chalcone, under catalysis by various enzymes, includes isomerases, hydroxylases, oxidoreductases. And post-modification enzymes, such as glycosyltransferases and methyltransferases, and acyltransferases, are then transformed into different phenylpropanoid products [17].

However, the development of industrial-scale processes for phenylpropanoid production is still facing serious problems. However, no applicable process for high production metrics have been established.The fermentation of some phenylpropanoids often requires complex culture conditions and processes to improve product titers [18, 19]. During the fermentation process of some phenylpropanoids such as raspberry ketone, it is difficult to develop an applicable bioconversion process to enrich biomass [20, 21]. It is also reported that the production of some phenylpropanoids is significantly reduced in glucose media [22, 23]. This makes fermentation procedures significantly more difficult and expensive when translated to large-scale processing.

Fatty acids (FAs) can serve as ideal alternative biomass resources with several advantages. Important metabolic precursor acetyl-CoA can be obtained from fatty acids through β-oxidation without carbon loss. This allows several products to be produced with high theoretical yields. Furthermore, the β-oxidation of fatty acids can release a large amount of reducing power essential for the synthesis of target products [24, 25]. Fatty acids materials can be obtained from various low-cost industrial and domestic wastes and industrial byproducts [26]. For example, when obtained from palm industry byproducts, the average carbon price per ton of palmitic acid is about $35 per ton of C (calculated from $570 per ton of palmitic acid extract), which is much lower than the average carbon price for glucose ($46 per ton of C, calculated from $275 per ton of glucose) [27]. In previous studies, we have successfully developed efficient routes to produce target chemicals, such as 3-hydroxypropionic acid and lycopene, in high yields from fatty acids [28, 29].

**Fig. 1:**
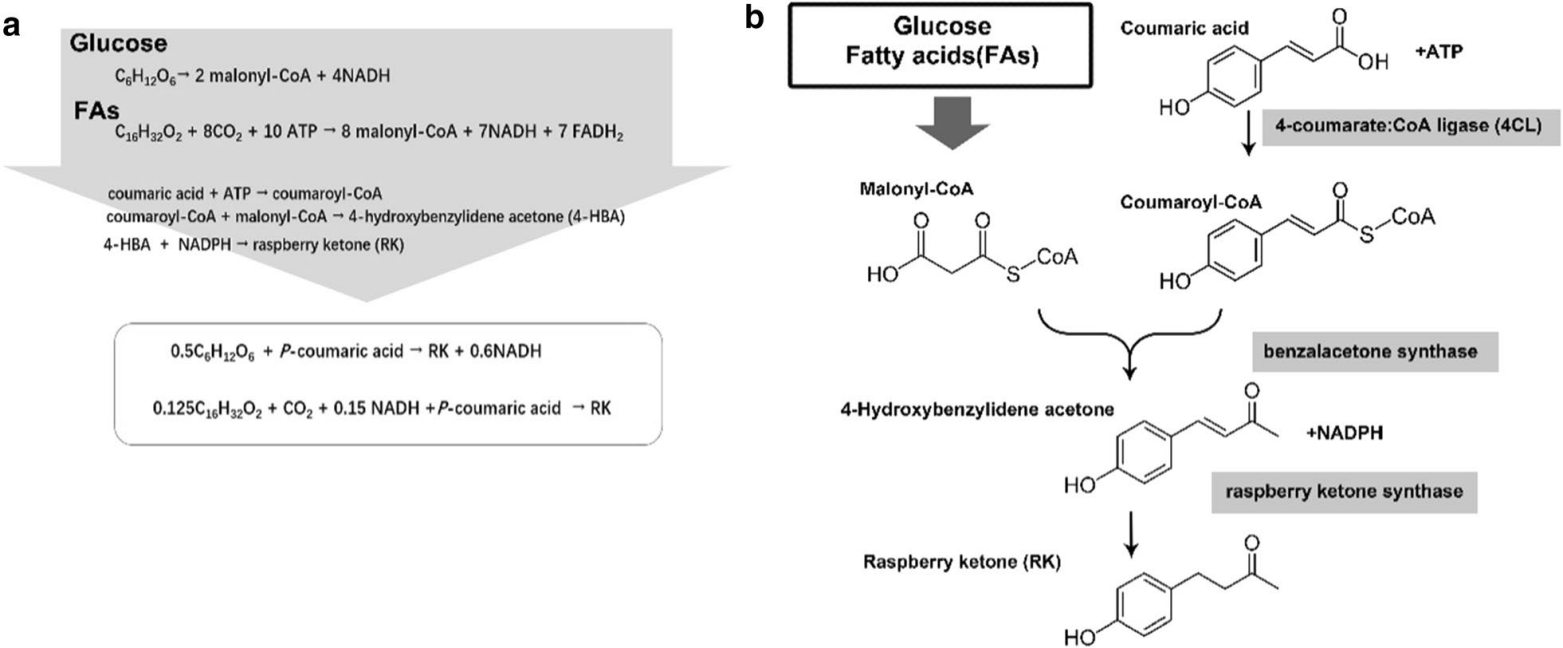
Production route of raspberry ketone (RK) from glucose, and fatty acids (FAs). **a** Equations from different carbon sources to RK; equations in the box show overall theoretical stoichiometry. **b** Enzymes involved in RK biosynthesis pathway

In this study, we sought to develop an efficient route for phenylpropanoid production using fatty acids as the feedstock. Typical phenylpropanoid raspberry ketone was used as the target product to develop a biosynthesis pathway for phenylpropanoid production from fatty acids. The pathway from fatty acids to phenylpropanoids has several advantages. Fatty acids could generate malonyl-CoA precursors for raspberry ketone biosynthesis with higher theoretical yield than glucose (Fig. 1). Bioproduction of raspberry ketone from fatty acids also might provide a solution to the bioprocess problem when using glucose feedstock. In this study, an efficient bioconversion process for raspberry ketone production was developed. Furthermore, to facilitate heterogeneous gene regulation and develop an ideal fermentation process, we systematically constructed a promoter system for use under fatty acids conditions. This study, for the first time, developed an efficient tools and protocols for phenylpropanoid production via fatty acids feedstock routes.

## Results

### Development and optimization of raspberry ketone biosynthesis module

**Fig. 2:**
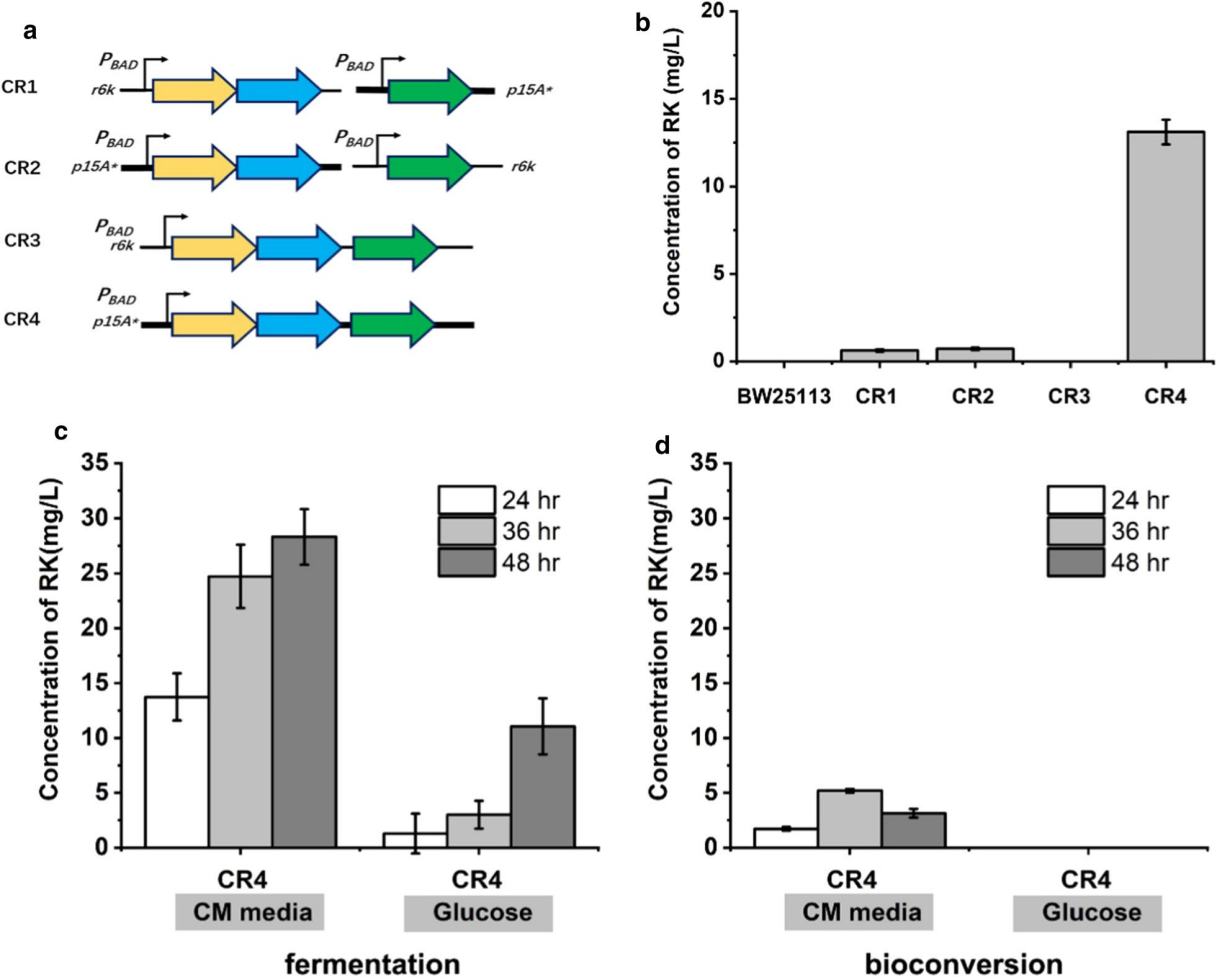
Strains optimization and RK production using a different strategy. **a** Construction of different RK strains. Thick lines indicate medium-copy-number plasmids; thin lines indicate low-copy-number plasmids; yellow arrows represent RiRZS1; blue arrows represent RpBAS, and green arrows represent At4CL1. **b** RK production by five different strains containing different plasmids singly or in combination. One-step fermentations were performed at 30 °C and 200 rpm for 24 h. CM medium containing 5 mM *p*-coumaric acid was used for RK production. **c** RK production by one-step fermentation by strain CR4. CM medium and M9-glucose medium containing 5 mM *p*-coumaric acid was used for RK production. One-step fermentations were performed at 30 °C and 200 rpm for 48 h. **d** RK production by bioconversion by strain CR4. CR4 was induced in the shake flasks containing CM medium at 30 °C and 200 rpm for 16 h. Cells were harvested, then suspended in CM medium or M9-glucose medium containing 5 mM *p*-coumaric acid (OD_600_ = 30). Bioconversions were performed at 30 °C and 200 rpm for 48 h

First, 4-coumarate-CoA ligase (4CL), benzalacetone synthase (BAS), and raspberry ketone reductase (RZS) were introduced and overexpressed in *E. coli* strains to develop a raspberry ketone biosynthesis module. Genes of At4CL1 from *Arabidopsis thaliana*, RpBAS from *Rheum palmatum*, and RiRZS1 from *Rubus idaeus* [21] were code optimized and constructed into either medium-copy-number p15A-derived plasmids or low-copy-number r6k-derived plasmids. Different plasmid combinations were transformed into *E. coli* BW25113. After one-step fermentation using complex enriched medium (CM medium, see materials and methods) for 24 h, strain CR1 containing pLB1a-RB and pYB1s-4CL could produce raspberry ketone with a concentration of 1.25 ± 0.1 mg/L, while strain CR2 containing pLB1a-4CL and pYB1s-RB could produce a raspberry ketone concentration of 1.03 ± 0.1 mg/L. In contrast, when RiRZS1, RpBAS, and At4CL1 were co-expressed in a single low-copy-number r6k-derived plasmid (pLB1a-RB4, strain CR3), Raspberry ketone production was not detected. This result implied that AT4CL1 expression was a key factor influencing raspberry ketone production. Consistent with these results, further replacing the r6k-derived plasmid with a medium-copy-number p15A-derived plasmid (pYB1s-RB4, strain CR4) resulted in a raspberry ketone concentration of 13.1 ± 0.2 mg/L (Fig. 2b).

Raspberry ketone production of CR4 was then carried out with different fermentation time periods. It was shown CR4 could produce raspberry ketone with 28.3 ± 2.5 mg/L after fermentation for 48 h in CM medium. However, only 11.1 ± 2.5 mg/L raspberry ketone was produced in glucose medium (48 h) (Fig. 2c). The strategy of whole-cell bioconversion using harvested cells is usually associated with high production titers. Thus, a two-step bioconversion experiment was carried out: cells enrichment and protein induction were carried out in CM medium first. Then cells were harvested and resuspended for bioconversion for the production of raspberry ketone. However, the raspberry ketone titers were very low when using bioconversion strategy in both CM medium (5.2 ± 0.2 mg/L) and glucose (0 mg/L) (Fig. 2d). It was shown *p*-coumaric acid was hardly consumed during bioconversion. This implied the biosynthesis pathway of raspberry ketone was not active in this condition.

### Development strains for raspberry ketone production from fatty acids feedstock

**Fig. 3:**
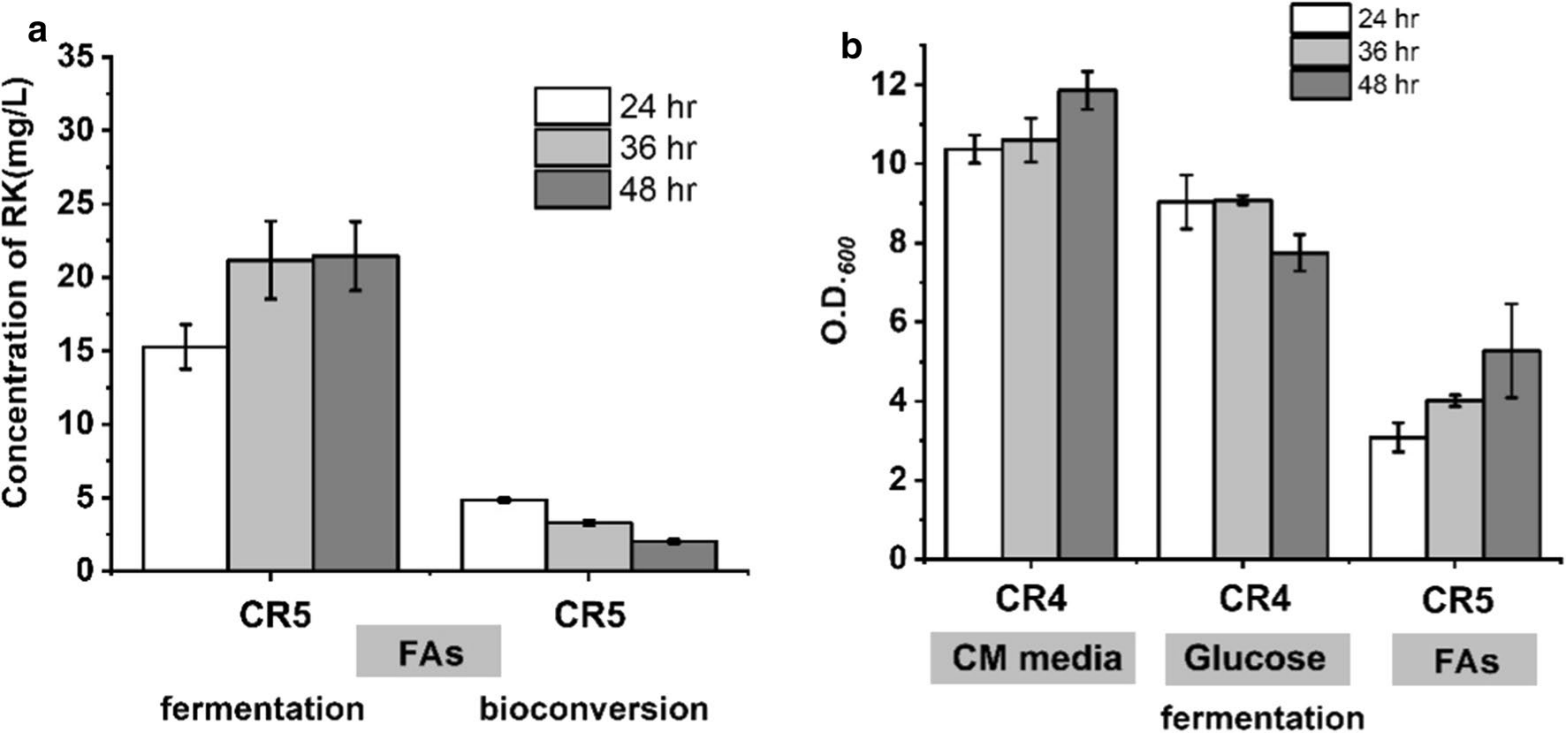
**a** RK production by CR5 strain in FAs medium using both fermentation and bioconversion strategy. M9-FAs medium containing 5 mM *p*-coumaric acid was used for fermentation. CM medium was used for bioconversion. CR5 was induced first in CM medium and harvested, then suspended in CM-FAs medium containing 5 mM *p*-coumaric acid (OD_600_ = 30). Fermentations and bioconversion were performed at 30 °C and 200 rpm. **b** Comparison of biomass in the different fermentation medium. CM medium, M9-glucose, and M9-FAs medium containing 5 mM *p*-coumaric acid were used for fermentation. Fermentations were performed at 30 °C and 200 rpm for 48 h

The raspberry ketone biosynthesis module was then transformed into a fatty acids-utilization host to develop the fatty acids route for raspberry ketone production. The degradation process of fatty acids is generally carried out by β-oxidation pathway. Taking palmitic acid as an example, long-chain fatty acids enter the cells through outer membrane transport protein(FadL) and are then activated to fatty acyl-CoA by fatty acyl-CoA synthetase(FadD). After entering the mitochondrial matrix, it undergoes multiple cycles under the catalysis of fatty acid β-oxidase system, and acetyl-CoA and fatty acyl-CoA are produced in each cycle. Acyl-CoA produces acetyl-CoA under the action of acyl-CoA dehydrogenase (FadE) and ketoacyl-CoA thiolase (FadAB). The pYB1s-RB4 plasmid was introduced into fatty acids utilization chassis cell FA09 to develop strain CR5 [28, 30–32]. First, one-step fermentation strategy was used to produce raspberry ketone using fatty acids as feedstock. It was shown that the titer of raspberry ketone was about 21.5 ± 2.4 mg/L after fermentation for 48 h, which was a little lower than that produced in CM medium. However, the titers were still found to be very low when the whole-cell bioconversion strategy was used (Fig. 3a). To assess the fermentation process in a different medium, the biomass of strains was recorded. It was shown that the biomass of strains during fermentation in fatty acids was much lower than in both CM medium and glucose (Fig. 3b). This result indicated that the fermentation conditions in the fatty acids medium should be optimized.

### Efficient raspberry ketone production by condition optimization

**Fig. 4:**
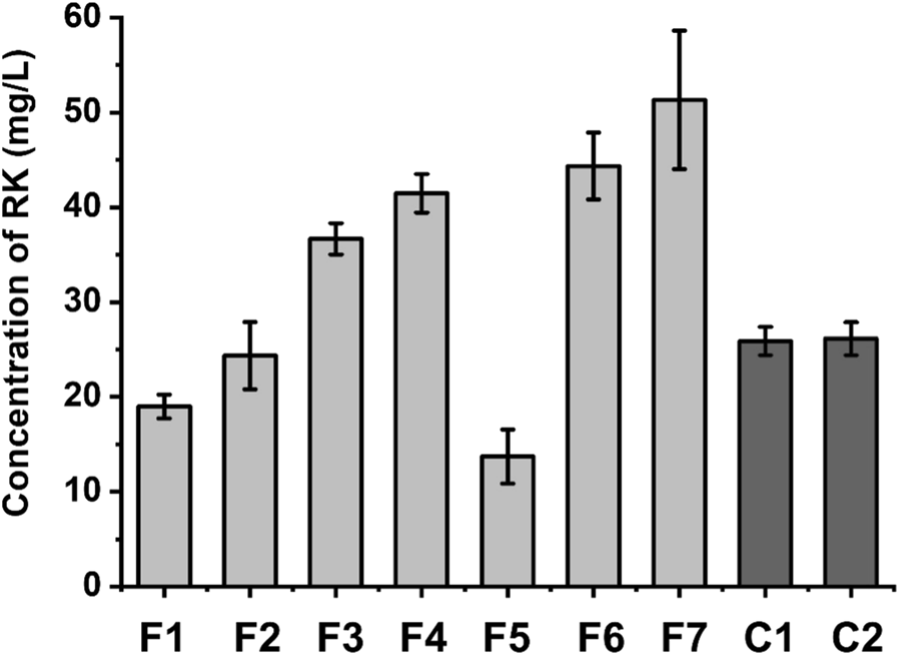
RK production under different fermentation conditions for CR5. MCM medium containing 5 mM *p*-coumaric acid was used for F1-F7 fermentation. The concentrations of different compositions used were: F1: none; F2: 0.2% FA; F3: 0.5% FA; F4:1% FA; F5: 1%FA + 0.5%glucose; F6: 1%FA + 0.5% glycerol; F7: 1%FA + 1% glycerol. Fermentations were performed at 30 °C and 200 rpm for 24 h. MCM7 medium was used for C1 and C2 bioconversion. C1: cells were induced by MCM7 medium and harvested, then suspended in MCM7 medium containing 5 mM *p*-coumaric acid (OD_600_ = 30); C2: Cells from CR5 cultured in MCM7 medium were harvested and suspended in MCM7 medium (OD_600_ = 30). Bioconversions were performed at 30 °C and 200 rpm for 24 h

Next, we sought to enhance cell growth and performance during fermentation in fatty acids. The fermentation medium was optimized by added different fatty acids compositions in the modified CM medium (MCM medium). The results indicated the biomass of all strains were similar to that in CM medium (about OD_600_ = 9–11). Raspberry ketone titers of strain CR5 gradually increased with increasing concentrations of fatty acids and reached 41.5 ± 2.0 mg/L in 1% fatty acids after fermentation for 24 h (Fig. 4f2–f4). Additional 1% glucose significantly decreased raspberry ketone titers to 13.7 ± 2.9 mg/L (Fig. 4f5). On the contrary, an additional 0.5% and 1% glycerol further increased raspberry ketone titers to 44.4 ± 3.5 and 51.3 ± 7.3 mg/L (named as MCM7 medium), respectively (Fig. 4f6–f7). Nevertheless, the optimized fatty acids fermentation medium was also suitable for the bioconversion strategy. Cultured cells of CR5 strain were resuspended in MCM7 medium with a starting OD_600_ = 30, and bioconversion was then carried out. Raspberry ketone titer of CR5 could reach 25.9 ± 1.5 mg/L after bioconversion for 24 h (Fig. 4c1). A similar titer (26.1 ± 1.8 mg/L) was also obtained when using cells harvested from CR5 cultured in MCM7 medium (Fig. 4c2).

### Screening and optimizing fatty acids response promoters

**Fig. 5:**
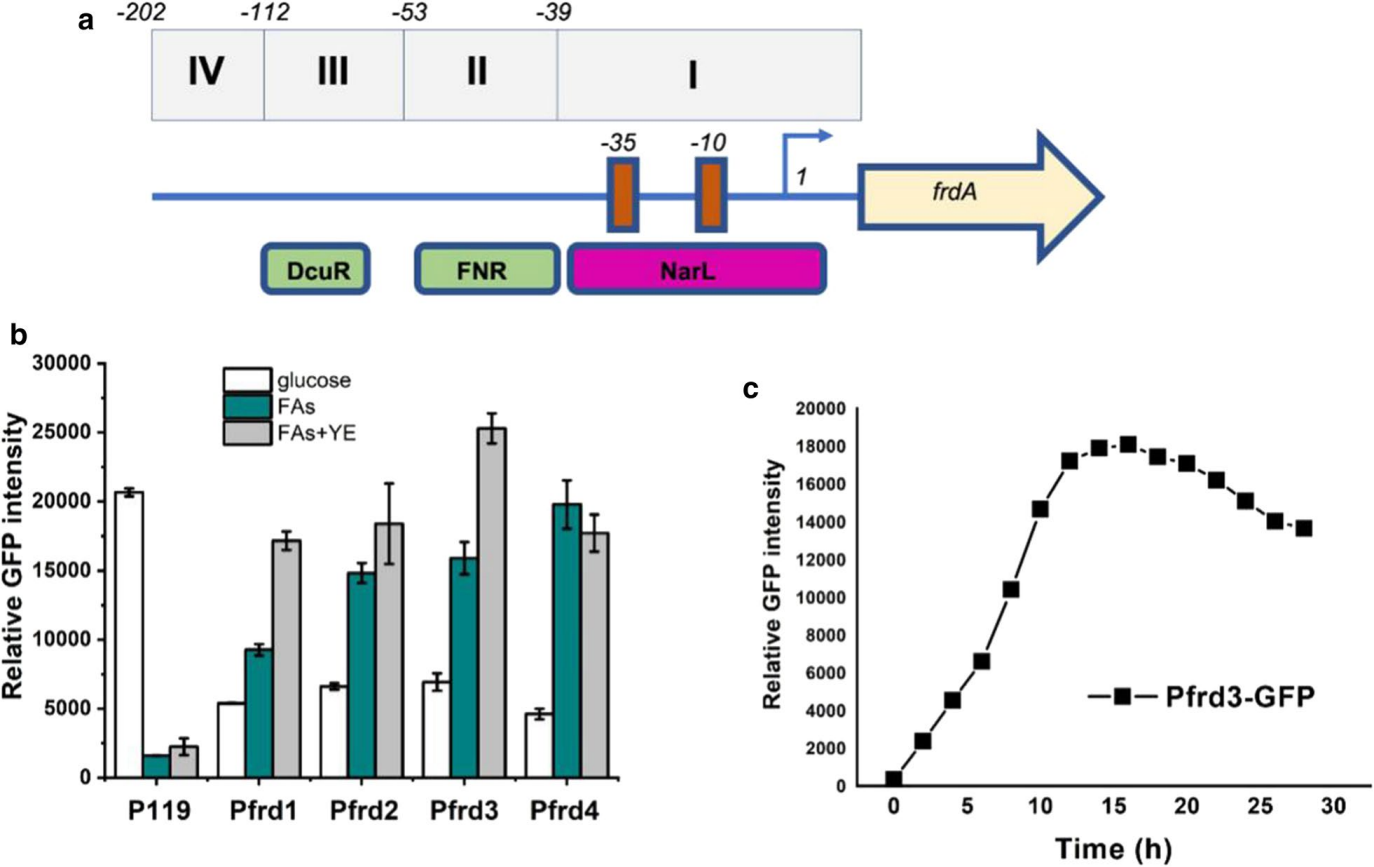
Screening FAs response promoters. **a** Length of truncations of the native frdA promoter. Symbols are grey filled rectangle: four truncated promoters; red filled rectangle: -10 region and -35 region of frdA gene; yellow rightwards arrow: frdA gene. **b** Comparison of the relative GFP intensity of different truncated promoters under different substrates. Growth in the M9 medium containing different carbon sources (1% glucose, 1%FA, 1% FAs A and 0.03% yeast extract) for 24 h. The control for these experiments was P119. **c** The relative GFP intensity of the Pfrd3 promoter at different times. Growth in the M9 medium containing 1% FAs and 0.03% yeast extract

According to above results, raspberry ketone titer was significantly increased by both fermentation and bioconversion in the optimized FA medium. This indicated fatty acids medium provided necessary factors related to raspberry ketone biosynthesis pathways. Considering that the expression of key enzymes, such as AT4CL1, are key limit factors for raspberry ketone production, expression of these enzymes should be fine regulated in response to the change of condition. Therefore, expression under promoters induced by fatty acids compositions provides a superior strategy to control the expression of key pathway enzymes. Then, we conducted promoter mining work to screen candidate promoters in response to fatty acids condition. We mainly focused on promoters involved in TCA cycles. This was because the expression of genes within TCA cycle was significantly enhances in fatty acids condition [28]. TCA-related promoters were constructed using green fluorescent protein (GFP) as reporter. The GFP intensity of different promoters was then analyzed after culture in fatty acids medium. Under several TCA-related promoters, GFP intensity increased in fatty acids conditions (data not shown). It was notable that the GFP intensity significantly increased under FA conditions using promoters PfrdA. Then four promoters with different truncations were designed (Fig. 5a). Different conditions were then investigated to determine the influence on the induction profiles of these promoters (Fig. 5c). The results indicated that GFP intensity using all promoters was significantly increased in fatty acids condition. Furthermore, additional 0.03% YE could further improve the GFP expression. The peak of GFP intensity occurred about 13 h after cultured in fatty acids condition.

### Modifying different modules using FA-induced promoters

**Fig. 6:**
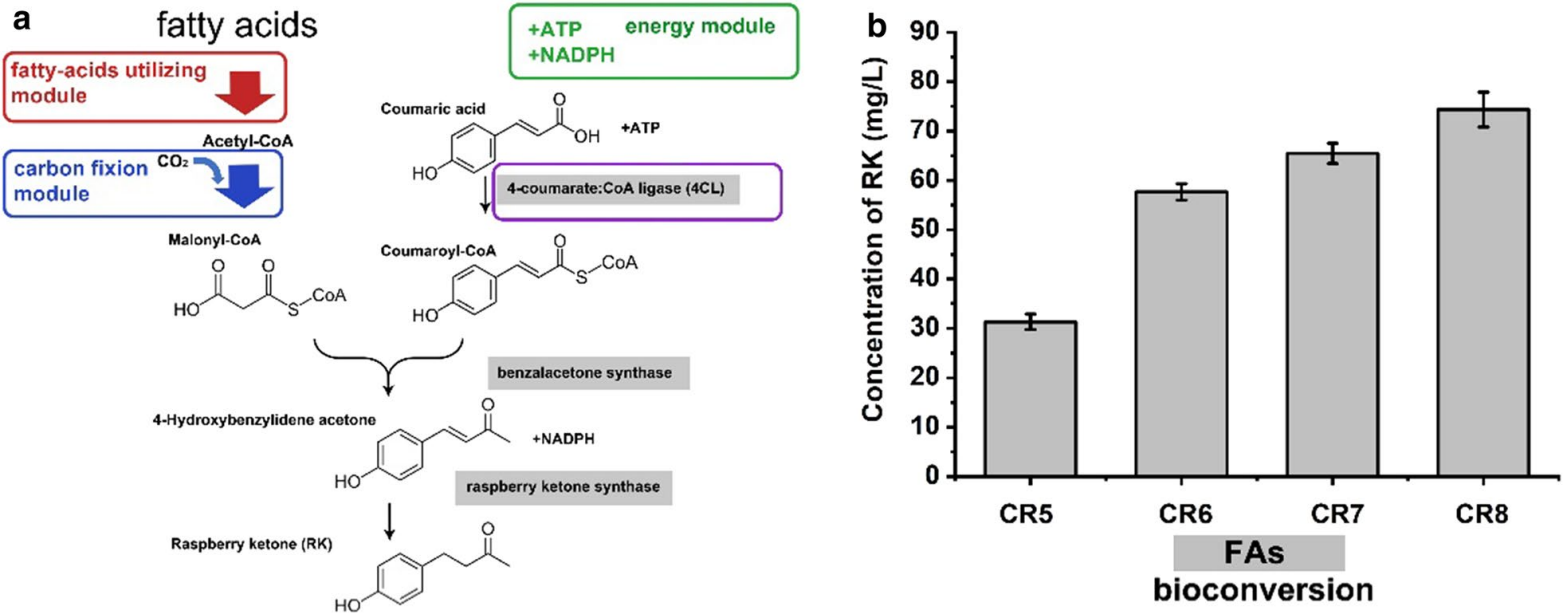
Metabolic engineering for RK production from FAs and RK production by different strains. **a** RK biosynthesis pathway from FAs; **b** RK production by different strains. Bioconversions were performed at 30 °C and 200 rpm. A palmitic acid concentration of 1% was used for RK production

Then we carried out genetic modification using above promoters to develop strains that were suitable for bioconversion strategy. First, an additional copy of the AT4CL1 gene was expressed under the control of Pfrd3 and chromosomally inserted at the poxB site, resulting in new strain CR6. Raspberry ketone production by CR6 was about 57.7 ± 1.7 mg/L after bioconversion for 24 h, which was much higher than that by CR5 (31.3 ± 1.5 mg/L) (Fig. 6). In *E. coli*, Type II fatty acid synthase (FAS) was responsible for FA production from acetyl-CoA and subsequent elongation using malonyl-acyl carrier protein (ACP) [33]. We attempted to increase the intracellular malonyl-CoA level by disrupting the competitive pathways. The fatty acids biosynthesis pathway is a major pathway for malonyl-CoA consumption. Thus, *fabB* gene encoding β-ketoacyl-[acyl carrier protein] synthases (fabB) that catalyze the condensation reactions of long-chain fatty acid synthesis should be knocked-out. Meanwile, studies have also been shown that the introduction of cyanobacteria carbon concentration mechanism (CCM) in *E. coli* could increase the concentration of inorganic carbon in the cell and further increase the malonyl-CoA supply, improved the production of 3- hydroxypropionic acid effectively [9, 27]. Therefore, the bicarbonate transporter (BT) gene from *Synechococcus sp.* and the carbonic anhydrase (CA) gene from *Anabaena sp. PCC 7120* were fused and chromosomal expressed by inserting at fabB site under the control of Pfrd3 promoter, obtained the strain CR7, and the final RK concentration reached 65.5 ± 2.0 mg/L.

Furthermore, RK reductase (RiRZS1) was NADPH-dependent, and the β-oxidation of fatty acids produces a large amount of NADH [34]. We attempted to further accumulate RK by regulating the conversion of NADH to NADPH. The tetramer [PntB]_2_[PntA]_2_ encoded by pntAB, the complex was a membrane-bound pyridine nucleotide transhydrogenase, which has a higher affinity for NADH and a weaker affinity for NADPH, so the enzyme mainly catalyzed the conversion of NADH into NADPH. Thus, additional pntAB under the regulation of Pfrd3 promoter was introduced to obtain strain CR8, which further increased the yield by 20% to 74.4 ± 3.5 mg/L, showing that NADPH had a considerable influence on RK synthesis.

### Efficient production of raspberry ketone by repeat bioconversion process

The bioconversion conditions were further optimized to achieve higher raspberry ketone production titers. Biomass during bioconversion was investigated at 10,20,30, and 40 O.D. It was shown the higher titer was obtained at 30 O.D. biomass (Additional file 1: Fig. S2). Then the speed of rotation was further optimized. Raspberry ketone titer with 93.3 ± 2.4 mg/L could be observed at an optimized condition, 220 rpm, 30 O.D biomass, bioconversion for 30 h.

**Fig. 7:**
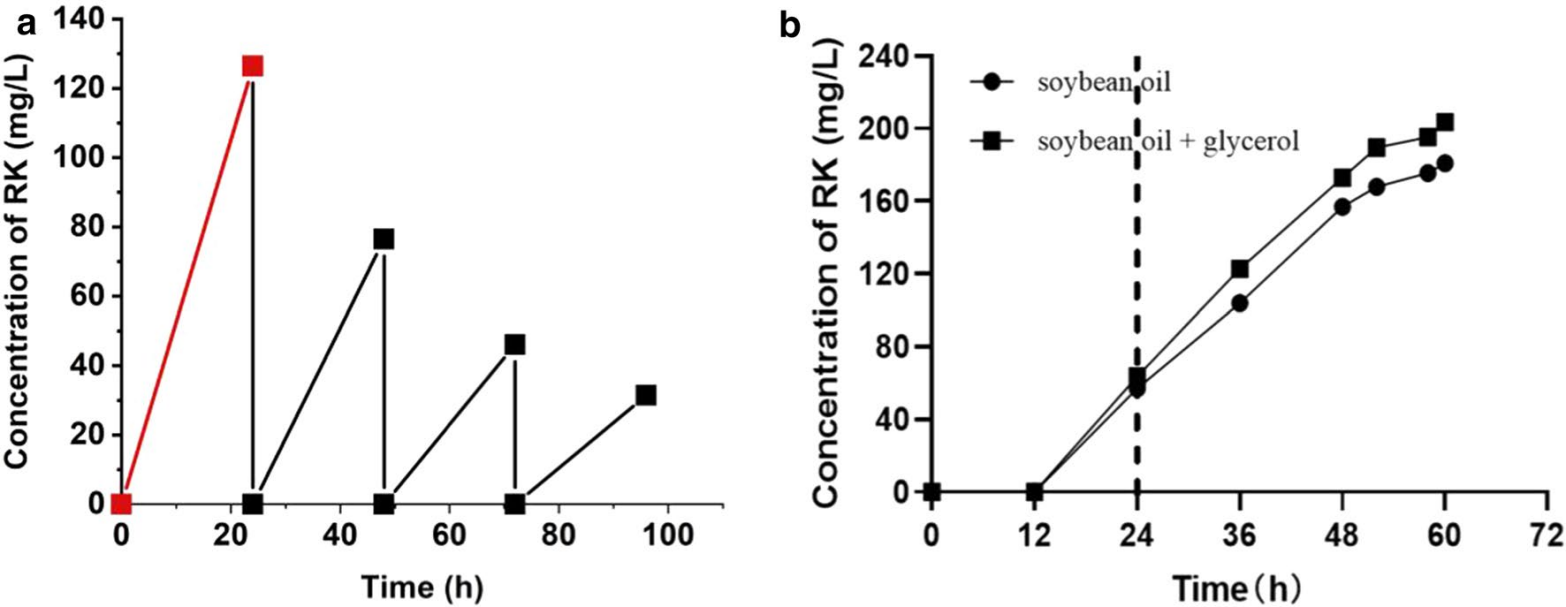
A. RK production by repeat bioconversion process. Red line indicates induction and cell growth stage. Bioconversion were performed at 30 °C and 220 rpm. A palmitic acid concentration of 1% was used for RK production. B. Fed-batch bioconversion in 1-L bioreactors for RK production using soybean oil or soybean oil + glycerol as substrate. The left side of the dotted line represents the cell growth and protein induction phase

Based on CR8, a continual fermentation process was conducted. First, CR8 strain was cultured and fermentation in MCM7 medium for 24 h, during which about 126.4 mg/L raspberry ketone could be produced. Then the cells were collected by centrifugation, resuspended in the MCM7 medium at 30 O.D. After bioconversion for additional 24 h, 76.4 mg/L raspberry ketone could be produced. The above operation was repeated three times. The raspberry ketone titers were 46.1 and 31.4 mg/L for the second and third bioconversion, respectively (Fig. 7a).

Furthermore, bioconversion was carried out in a 1-L bioreactor. RK production of strain CR8 was investigated by feeding with soybean oil as a single substrate (Fig. 7b and Additional file 1: Fig. S3). CR8 strain was first cultivated in CD medium for 24 h. Then, soybean oil was fed in batches at 1 g/L/h. Soybean oil and glycerol were used as mixed substrates and fed according to a 1:1 carbon ratio, the total carbon of the mixed feed was remained the same as the glycerol feed. P-coumaric acid was fed in batches at 2.5 mmol /L/12 h The final RK concentration was about 203 mg/L when using mixed substrates reached (Fig. 7b).

## Discussion

Phenylpropanoids, such as resveratrol and naringenin, are a class of natural products widely found in plants and microorganisms, and have important applications in medicines, cosmetics, and health products [6]. To date, no practical industrial process for the microbial production of phenylpropanoids have been developed. Whole-cell bioconversion is a more efficient technique route for production of not only raspberry ketone and other phenylpropanoids, but also other diverse bioproducts. However, it is difficult to develop an efficient bioconversion process for raspberry ketone production previously. Raspberry ketone and other phenylpropanoids synthesis require *p*-coumaric acid or cinnamic acid as precursor, which are derived from aromatic amino acids. After activation by CoA, *p*-coumaric acid or cinnamic acid is polymerized with different units of malonyl-CoA to form the final target product. However, similar to previous reports, our study found that 4CL-mediated activation of *p*-coumaric acid was the critical rate-limiting step [21]. To our surprise, when glucose was used as the raw material, the raspberry ketone yield was low. Similar phenomenon is also reported in the production of some other phenylpropanoids [21, 35]. The underling mechanism of the glucose inhibitory effect on bioproduction is still to be explored. There might be several reasons for this. First, the function or expression of key enzymes is limited under glucose conditions. Second, the level of some important factors or metabolites is not sufficient for the biosynthsis pathway. The precursor *p*-coumaric acid is significantly accumulated when glucose was used, suggesting that fine regulation of 4CL expression and CoA levels in cells was a key factor in this synthesis. To remove this bottleneck and achieve efficient expression of enzyme 4CL in fatty acids raw materials, we systematically screened promoters that can be induced by fatty acids. The results showed that relevant promoters could be used in our strains to successfully increase the raspberry ketone production.

Malonyl-CoA is among the most important metabolites, and can serve as a basic building block for fatty acids biosynthesis. Therefore, malonyl-CoA is the key precursor of diverse fatty-acid-derived compounds, including biofuels [36]. Malonyl-CoA is also a precursor for the microbial synthesis of many pharmaceutically interesting polyketides and natural products, such as phenylpropanoids [37]. Fatty acids are a promising resource for the biosynthesis of malonyl-CoA via carboxylation of acetyl-CoA, which can be efficiently obtained from the β-oxidation of fatty acids. As fatty acids are an easily obtained feedstock, they are ideal raw materials for novel production routes to natural products [28]. In this study, a novel biosynthesis strategy to produce raspberry ketone (a representative phenylpropanoid) from fatty acids raw materials was reported for the first time. These results demonstrate a novel technical route for the production of similar compounds.

Furthermore, establishing an efficient induction and fermentation process might be key to achieving a high-level fermentation strategy for phenylpropanoid production. We solved this problem through cooperative utilization of multiple carbon sources and fine-tuning of key enzyme expression. Further studies will focus on using different FA resources as raw materials. In summary, we conducted systematic metabolic engineering of *E. coli* strains to produce raspberry ketone from fatty acids. A module for raspberry ketone synthesis was constructed by introducing 4CL, BAS, and RZS. Then the raspberry ketone synthesis module was combined with the fatty acids utilization module to construct the engineered strain for raspberry ketone production from fatty acids. We systematically screened the fatty acid-inducible promoters. The expression of key enzymes under Pfrd3 promoter contributed to *p*-coumaric acid activation, increasing the supply of precursors and NADPH. Finally, the strain CR08 produced raspberry ketone by using multiple carbon sources, and produced 180.94 mg/L raspberry ketone by using soybean oil in a 1-L fermentation. This study will help use cheaper raw materials to produce not only raspberry ketone, but also other important flavonoid compounds.

## Materials and methods

### Strains and reagents

DNA polymerases, Taq Mix, T4 DNA ligase, Gibson Assembly® One Step Cloning Kit were purchased from Vazyme Biotech Co. Ltd. (Nanjing, China). Restriction enzymes were purchased from New England Biolabs (USA). Plasmid Mini Kits, PCR Clean-Up Kits, and Gel Extraction kits were all obtained from Omega Bio-Tek Co. Ltd. (USA). *P*-coumaric acid and raspberry ketone was purchased from Solarbio Biochemical Co. Ltd. (Shanghai, China). All other regular chemicals were purchased from ShengGong Biochemical Co. Ltd. (Shanghai, China). The primer synthesis and gene sequencing were done in GENEWIZ, Inc. (Beijing, China).

The *E. coli* strain DH5α was used for plasmids construction, BW25113/F′ were used for protein expression and raspberry ketone production, respectively. Luria–Bertani (LB) medium was used for all molecular construction experiments and strain culture. Strains containing temperature-sensitive plasmids pKD46, pCP20, and pSB1s-cre were cultured at 30 °C, and the remaining strains were cultured at 37 °C without special instructions.

Luria–Bertani (LB) medium containing 10 g/L tryptone, 5 g/L yeast extract, and 10 g/L NaCl was used to grow *E. coli* cells unless otherwise stated.  1× M9 salts containing 12.8 g/L Na_2_HPO_4_∙7H_2_O, 3 g/L KH_2_PO_4_, 0.5 g/L NaCl, 1 g/L NH_4_Cl was used to bioconversion. M9-FAs medium containing 1 × M9 salts, 2 mM MgSO_4_, 10 g/L palmitic acid, M9-FAs + YE (supplemented with YE), M9-glucose (10/L glucose instead of palmitic acid) were used to screen FA response promoters. CM medium containing 10 g/L tryptone, 5 g/L yeast extract, 5 g/L glycerol, 0.5 g/L glucose, 25 mM Na_2_HPO_4_, 25 mM KH_2_PO_4_, 50 mM NH_4_Cl, 5 mM Na_2_SO_4_, 2 mM MgSO_4_ and trace elements was used for one-step fermentation and cell preparation for bioconversion. Modified CM medium containing 1 × M9 salts, 5 g/L tryptone, 2 mM MgSO_4_ and trace elements was used to one-step fermentation and bioconversion. When necessary, the antibiotics were used (ampicillin, 100 μg/mL; streptomycin, 50 μg/mL; and kanamycin, 50 μg/mL).

### Construction of plasmids and strains

All strains and plasmids used in this study are shown in Table 1. All primers are listed in Additional file 2: Table S1. At4CL1 (GenBank ID: AAA82888.1) from *Arabidopsis thaliana*, RpBAS (GenBank ID: AAK82824.1) from *Rheum palmatum*, and RiRZS1 (GenBank ID: JN166691) from *Rubus idaeus* were codon-optimized for *E. coli* expression [17, 38–40].

**Table 1:** Strains and plasmids used in this study

Strain	Genotype	Source
*E. coli* BW25113/F^׳^	*rrnBT*14 Δ*lacZWJ*16 *hsdR*514 Δ*araBADAH*33 Δ*rhaBADLD*78 [F^׳^ *proAB lacIqZ*ΔM15 *Tn*10 (*Tetr*)]	CGSC
FA09	*E. coli* BW25113/F^׳^, Δ*fadR,* P_CPA1_*-fadD,* P_119_*-fadL,* Δ*sthA,* P_CPA1_*-pntAB*	[28]
CC1	FA09, Δ*poxB::* P_frd3_-*AT4CL1*	This study
CC2	CC1, Δ*fabB::* P_119-_BT-CA	This study
CC3	CC2, P_frd3_*-pntAB*	This study
CR1	*E. coli* BW25113/F^׳^ carrying pLB1a-RB and pYB1s- 4CL	This study
CR2	*E. coli* BW25113/F carrying pYB1s-RB and pLB1a-4CL	This study
CR3	*E. coli* BW25113/F^׳^ carrying pLB1a-RB4	This study
CR4	*E. coli* BW25113/F^׳^ carrying pYB1s-RB4	This study
CR5	FA09 carrying pYB1s-RB4	This study
CR6	CC1 carrying pYB1s-RB4	This study
CR7	CC2 carrying pYB1s-RB4	This study
CR8	CC3 carrying pYB1s-RB4	This study

Plasmid	Description	Source
pLB1a	araBAD promoter, R6k ori, Amp^r^	Our laboratory
pYB1s	araBAD promoter, p15A ori, Str^r^	Our laboratory
pKD46	Temperature-sensitive vector carrying Red recombinase, Amp^r^	[42]
pSB1s-Cre	Temperature-sensitive vector carrying Cre recombinase, Str^r^	Our laboratory
pCP20	Temperature-sensitive vector carrying FLP recombinase, Amp^r^	[42]
pLB1a-RB	pLB1a containing *Rubus idaeus* RZS1 gene and *Rheum palmatum* BAS gene	This study
pLB1a-4CL	pLB1a containing *Arabidopsis thaliana* 4CL1 gene	This study
pYB1s-RB	pYB1s containing *Rubus idaeus* RZS1 gene and *Rheum palmatum* BAS gene	This study
pYB1s- 4CL	pYB1s containing *Arabidopsis thaliana* 4CL1 gene	This study
pLB1a-RB4	pLB1a containing *Rubus idaeus* RZS1 gene, *Rheum palmatum* BAS gene and *Arabidopsis thaliana* 4CL1 gene	This study
pYB1s-RB4	pYB1s containing *Rubus idaeus* RZS1 gene, *Rheum palmatum* BAS gene and *Arabidopsis thaliana* 4CL1 gene	This study
pYfrd11s-GFP	frd1 promoter, p15A ori, Str^r^, GFP gene	This study
pYfrd21s-GFP	frd2 promoter, p15A ori, Str^r^, GFP gene	This study
pYfrd31s-GFP	frd3 promoter, p15A ori, Str^r^, GFP gene	This study
pYfrd41s-GFP	frd4 promoter, p15A ori, Str^r^, GFP gene	This study

Molecular cloning and genetic editing were performed using standard protocols. For knockout genes, a single knockout library stored in the laboratory was used to achieve integration through P1 phage infection [41]. pCP20 eliminated the resistance marker by identifying the FRT sites at both ends of the resistance marker gene. The plasmids pYB1s and pLB1a were previously constructed in our laboratory; the specific features were as follows: streptomycin and kanamycin resistance genes, araBAD promoter, multiple cloning sites, rrnB terminator, p15A, and R6k. Promoter replacement and gene insertion replacement used the gene-editing tool plasmids constructed in our laboratory as templates for amplification to obtain homologous recombination fragments. pSB1s-cre eliminated the resistance marker by identifying the lox66 and lox71 sites at both ends of the resistance marker gene.

### One-step fermentation conditions

When used CM medium for one-step fermentation, the recombinant strains were grown in CM medium to an OD_600_ of 0.4–0.6 at 30 °C, added 5 mM *p*-coumaric acid, and 2 g/L l-arabinose, then cultured for a given time. When used glucose for one-step fermentation, the recombinant strains were grown in M9 modified medium to an OD_600_ of 0.4–0.6 at 30 °C, added 2 g/L l-arabinose and induced overnight, supplemented with 10 g/L glucose, 5 mM *p*-coumaric acid, then cultured for a given time. When used fatty acids for one-step fermentation, the process was the same as the glucose for one-step fermentation process, except for supplementing 10 g/L fatty acids.

For condition optimization, the following methods were used to optimize the substrate: (1) different concentrations of fatty acids were added (2) 1% fatty acids and different concentrations of glycerol were used together with different concentrations of fatty acids (2) 1% fatty acids and 0.5% glucose.

### Bioconversion conditions

The induction conditions we changed according to the experiment in the study, cells were collected after induction by centrifugation at 6000×*g* for 10 min, washed twice with 0.85% NaCl solution, suspended in 3 mL bioconversion medium in a test tube containing 5 mM* p*-coumaric acid and different feedstocks, starting biomass of OD_600_ = 30(unless otherwise specified), growing at 30 °C, 200 rpm for 24 h. The bioconversion medium changed based on experimental conditions. The carbon sources glucose, glycerol, or fatty acids was also added based on the experimental conditions. In the process of optimizing the bioconversion conditions, the bioconversion starting biomass of OD_600_ was investigated at 10, 20, 30 and 40.

### Cell growth and fluorescence analysis

To compare the strength of FA response promoters, the GFP gene was placed under different promoters' control and introduced into a FA utilization chassis cell FA09. After overnight cultured, inoculated to M9-FAs medium, M9-glucose medium, and M9-FAs + YE medium, starting biomass of OD_600_ = 0.1, 37 °C, and 200 rpm culture for 36 h. GFP intensity was measured using the BioTek Synergy Mx enzyme marker (BioTek, Winooski, VT, USA). Excitation at 468 nm, emission at 512 nm, automatic gain.

Fatty acids emulsified in the medium were opaque emulsion, so OD_600_ could not be used directly to detect biomass. We mixed an equal volume of ethyl acetate with the bacterial solution, centrifugation at 6000 × g for 1 min, the fatty acid was sandwiched between the ethyl acetate and the medium, discarded all the supernatant, resuspended the cells with an appropriate amount of 0.85% NaCl solution, and monitored the biomass in OD_600_.

### Analytical methods

HPLC determined product concentration. A 500 μL sample was taken and mixed thoroughly with the same amount of absolute ethanol by vortexing 30 s. The sample was centrifuged at 8000 × *g* for 10 min and filtered with a 0.2 µm PES membrane filter (Jinteng, Tianjin, China).

The concentrations of raspberry ketone and *p*-coumaric acid were measured by high-performance liquid chromatography (HPLC, LC 20A LabSolutions, Shimadzu Corp., Kyoto, Japan) with an Agilent Extend-C18 3.5 μm column (4.6 × 250 mm). The column was at 35 °C, and a flow rate of 0.5 ml/min was used. Mobile phase A (65%) was water with 0.1% (v/v) formic acid; mobile phase B (35%) was acetonitrile. Raspberry ketone and *p*-coumaric acid were detected via DAD detection. The raspberry ketone was detected at 222 nm, *p*-coumaric acid was detected at 305 nm.

## Supplementary Information

**Additional file 1: Figure S1.** The relative GFP intensity of the Pfrd3 promoter at different OD conditions. **Figure S2.** OD optimization and rotation speed optimization. (A) Production comparison between different OD conditions. (B) Production comparison between different rotation speeds. **Figure S3.** Remaining soybean oil of Fed-batch fermentation in 1-L bioreactors for RK production.

**Additional file 2: Table S1.** The primers used in this study.

## Data Availability

Not applicable.

## Abbreviations

RK: Raspberry ketone
FAs: Fatty acids
4CL: 4-Coumarate-CoA ligase
BAS: Benzalacetone synthase
RZS: Raspberry ketone reductase
CM: Complex enriched medium
MCM: Modified complex enriched medium
TCA: Tricarboxylic acid
LB: Luria–Bertani
