# Supplementary material for: Efficient bioconversion of raspberry ketone in Escherichia coli using fatty acids feedstocks
Source: Microb Cell Fact. 2021 Mar 12;20:68. doi: 10.1186/s12934-021-01551-0 (PMC7953670; doi:10.1186/s12934-021-01551-0)
Supplement: Supplementary file 1 — Additional file 1: Figure S1. The relative GFP intensity of the Pfrd3 promoter at different OD conditions. Figure S2. OD optimization and rotation speed optimization. (A) Production comparison between different OD conditions. (B) Production comparison between different rotation speeds. Figure S3. Remaining soybean oil of Fed-batch fermentation in 1-L bioreactors for RK production. [file 12934_2021_1551_MOESM1_ESM.docx]

**Efficient bioconversion of raspberry ketone in *Escherichia coli* using fatty acids feedstocks**

**Chen Chang^#1,2^, Bo Liu^#2^, Yihong Bao^*1,4^, Yong Tao^*2,3^, Weifeng Liu^*2,3^**

1. College of Forestry, Northeast Forestry University, No.26 Hexing Road, Harbin, Heilongjiang Province, 150040, PR China
2. CAS Key Laboratory of Microbial Physiological and Metabolic Engineering, State Key Laboratory of Microbial Resources, Institute of Microbiology, Chinese Academy of Sciences, NO.1 Beichen West Road, Chaoyang District,Beijing, 100101, PR China
3. University of Chinese Academy of Sciences, NO.19A Yuquan Road, Shijingshan District, Beijing, 100049, PR China
4. Heilongjiang Key Laboratory of Forest Food Resources Utilization, No.26 Hexing Road, Harbin, Heilongjiang Province, 150040, PR China

# These authors contributed equally to this work.

* Corresponding authors.

[taoyong@im.ac.cn](mailto:taoyong@im.ac.cn)

[baoyihong@163.com](mailto:baoyihong@163.com)

[liuwfv@im.ac.cn](mailto:liuwfv@im.ac.cn)

Figure S1. The relative GFP intensity of the Pfrd3 promoter at different OD conditions.

Figure S2. OD optimization and rotation speed optimization. (A) Production comparison between different OD conditions. (B) Production comparison between different rotation speeds.

Figure S3. Remaining soybean oil of Fed-batch fermentation in 1-L bioreactors for RK production.
