# Supplementary material for: Efficient bioconversion of raspberry ketone in Escherichia coli using fatty acids feedstocks
Source: Microb Cell Fact. 2021 Mar 12;20:68. doi: 10.1186/s12934-021-01551-0 (PMC7953670; doi:10.1186/s12934-021-01551-0)
Supplement: Supplementary file 2 — Additional file 2: Table S1. The primers used in this study. [file 12934_2021_1551_MOESM2_ESM.docx]

**Table S1** The primers used in this study

| Primes | Sequence(5´-3´) |
| --- | --- |

| NCOI-R | GGTTAATTCCTCCTGTTAGC |
| --- | --- |
| XHOI-F | CTCGAGGGTAGATCTGGTACTA |
| NCOI-At4CL1-F | GCTAACAGGAGGAATTAACC ATGGCGCCGCAGGAGCAAGCGG |
| XHOI-At4CL1-R | TAGTACCAGATCTACCCTCGAG TTACAGGCCGTTCGCCAGTTTCGC |
| NCOI-RiRZS1-F | GCTAACAGGAGGAATTAACC ATGGCGAGCGGTGGCGAGATGCAG |
| RBS-RpBAS-F | AGGAGGAATTAAATGGCGACCGAGGAAATGAAG |
| XHOI-RpBAS-R | TAGTACCAGATCTACCCTCGAG TTAGCTAATAACCGGCACGCTACGC |
| RpBAS-R | TTAGCTAATAACCGGCACGCTACGC |
| RiRZS1-R | TTATTCACGGCTAACCACAACC |
| RBS-At4CL1-F | AGGAGGAATTAAATGGCGCCGCAGGAGCAAGCGG |
| pBAD-108F | CCATAGCATTTTTATCCATAAGATT |
| pBAD-124R | AGAAGCGGTCTGATAAAACAGAATT |
| 119-F | GCAGGGAGACCACAACGGTT |
| CPA1-F | ACGAAACAGCCTCTACAAAT |
| tac-F | ATAATGTGTGGAGGGAGACCA |
| kan-600R | TTTCCACCATGATATTCGGC |
